# Influence of the Separation of Prescription and Dispensation of Medicine on Its Cost in Japanese Prefectures

**DOI:** 10.5539/gjhs.v6n4p57

**Published:** 2014-04-08

**Authors:** Masayuki Yokoi, Takao Tashiro

**Affiliations:** 1 The Open University of Japan, Japan

**Keywords:** medicine, economic, multiple regression analysis

## Abstract

We studied how the separation of dispensing and prescribing of medicines between pharmacies and clinics (the “separation system”) can reduce internal medicine costs. To do so, we obtained publicly available data by searching electronic databases and official web pages of the Japanese government and non-profit public service corporations on the Internet. For Japanese medical institutions, participation in the separation system is optional. Consequently, the expansion rate of the separation system for each of the administrative districts is highly variable. The data were subjected to multiple regression analysis; daily internal medicines were the objective variable and expansion rate of the separation system was the explanatory variable. A multiple regression analysis revealed that the expansion rate of the separation system and the rate of replacing brand name medicine with generic medicine showed a significant negative partial correlation with daily internal medicine costs. Thus, the separation system was as effective in reducing medicine costs as the use of generic medicines. Because of its medical economic efficiency, the separation system should be expanded, especially in Asian countries in which the system is underdeveloped.

## 1. Introduction

Previous studies have examined the factors that influence medical expenditures. Hitiris and Posnett (1992) reported that the strong positive correlation between per capita health spending and gross domestic product (GDP) was confirmed by their analysis of data of the 20 OECD countries. Mastuda (1992) reported that the use of health examinations was negatively correlated with medical expenditures for the elderly for both in- and outpatient services. Nevertheless, a few studies have examined the influence of the separation of dispensing and prescribing medicines between pharmacies and clinics on medicine costs. The separation of dispensing and prescribing medicines between pharmacies and clinics (hereafter referred to as the “separation system”) has been common practice for many years in North American and European countries. In contrast, most Asian countries, notably China and Japan, have only just begun to introduce the separation system. The separation system was adopted in Japan because the government thought it would reduce medicine costs by decreasing the number of duplicate prescriptions and reducing the economic motive for prescriptions.

In Japan, the separation system became optional in the 1960s. This system involves separating the prescriber from the dispenser of medicines, both duties formerly carried out by the Japanese Ministry of Health, Labour, and Welfare (JMHLW). Proponents of the system have argued that it reduces medicine costs, but since its introduction, there have been conflicting reports about whether this system does, in fact, do so (Nakamura, Nagaki, Iizuka, & Fujii, 1989). Furthermore, although the Japanese government has facilitated the expansion of the separation system, many people doubt its cost effectiveness. Critics of the system blame it for increasing medical expenses throughout the country, particularly in times of financial crisis (Watanabe, 1996).

The economic effects of the separation system have rarely been studied with quantitative data because it is difficult to precisely compare the objects under study. Therefore, it is unclear whether the system promotes the proper prescription of medicines or reduces medicine costs (Nakamura, Nagaki, Iizuka, & Fujii, 1989; Watanabe, 1996). A few previous studies have examined the relationship between the separation system and medicine costs. The “Ueda model study” found that medicine charges per patient in the group that consumed 20–100% of all prescription medicines were approximately 20% lower than those of the group that consumed 0–20% (Nakamura et al., 1989). In this study, the changes in medicine charges due to the separation system were compared between those of Ota Ward in Tokyo, Haibara Ward in Shizuoka prefecture, Ueda City in Nagano prefecture, and Wakamatsu Ward in Kitakyushu City in Fukuoka prefecture. Although medicine charges increased by 20% in Haibara Ward due to the separation system, they decreased by 10–20% in the other three districts. However, because these studies are outdated, their findings do not provide robust support for the notion that the separation system is cost efficient.

More recently, Kinoshita et al. (2004) investigated the level of economic efficiency before and after the switch to dispensing medicines outside clinics. They reported that they could not reach any conclusion about whether the medicine costs under the separation system are lower than when medicine is prescribed and dispensed at hospitals. Furthermore, their report is based on a relatively small sample of 24 hospitals and clinics. Thus, it remains unclear how the separation system influences medicine costs. To address this, we conducted the present study, hypothesizing that the separation system has contributed to reduced daily medicine cost. Moreover, we attempted to verify the specific contribution of the separation system to reduced medicine cost.

## 2. Method

Japan has a universal national health insurance system. Consequently, all Japanese people belong to a specific public health insurance system. Therefore, by analyzing public insurance data, we can estimate the health and medical costs of the whole nation. Importantly, because of the frequent changes in the Japanese insurance system, it is difficult to evaluate the exact effect of each factor on medicine costs. Here, we examined the correlation between medicine costs per prescription and the factors believed to influence medical expenses by analyzing objective nationwide prefecture-level data derived from three sources: the Japanese Statistics Bureau of the Ministry of Internal Affairs and Communications (JSB), the JMHLW, and the Japan Pharmaceutical Association (JPA), a non-profit public service corporation.

**Figure 1:**
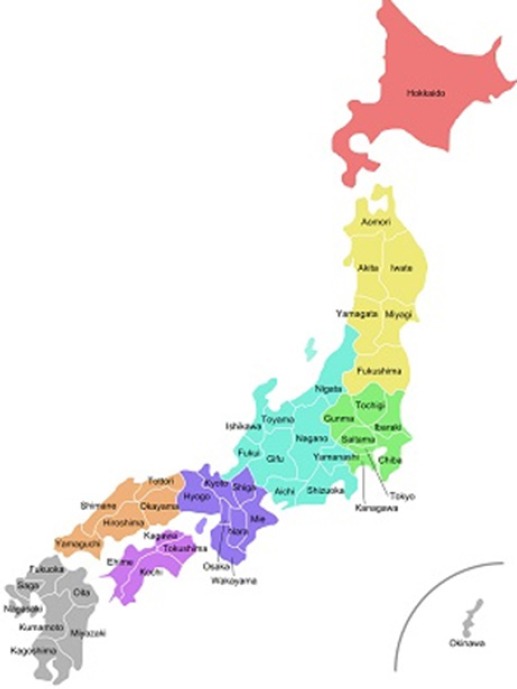
Japan’s 47 prefectures

Japanese prefectures are broad-based common municipal corporations. They divide Japan into 47 administration districts as shown in Figure 1.

In the present study, we examined the financial efficiency of separating pharmacies from clinics by evaluating data from each of Japan’s prefectures.

The result would be a clear understanding of the cost efficiency of the separation system. To do so, we performed a multiple linear regression analysis with daily medicine costs for internal use or other medicine costs per prescription as the dependent variables and the expansion rate of the separation system, the base ratio of generic medicine replaced with brand name medicine, and the proportion of people 75 years or older age in the population, as independent variables. We hypothesized that these variables would have a considerable influence on daily medicine costs, as explained in the following paragraphs.

Currently, while the separation system is optional for hospitals and clinics, its use is gradually increasing throughout the country. The separation of prescription and medicine sales enables doctors to refrain from prescribing specific name brand medicines.

Generic medicines can be sold only when the patent on the name brand of the pharmaceutical formula expires. The market price for generic medicines is much lower than that of brand-name medicines. Consequently, daily medicine prices should be lower in the areas that dispense more generic medicines than name brand medicines compared to the areas that dispense more name brand medicines than generic medicines.

Finally, medical expenses tend to increase as people age. In 2011, 33% of the total amount of money spent on medicines was for people 75 years or older (Ministry of Health, Labour and Welfare Insurance Bureau Security Research Division, 2011). Thus, we expected prefectures with a greater proportion of elderly people to have higher medical costs.

### 2.1 Dependent Variable

We obtained 2011 fiscal data on average medicine cost and dosage days per prescription for each prefecture from the JMHLW website (Ministry of Health, Labour, and Welfare Insurance Bureau Security Research Division, 2011). We then divided the average medicine charge per prescription by the dosage days per prescription to obtain the daily medicine cost.

Nationwide data on prescriptions are available from insurance applications for medical expenses received from pharmacies, 99.9% of which are currently covered by medical insurance. We could access these data freely because insurance applications for medical expenses from pharmacies have been made available on the JMHLW website since 2009.

### 2.2 Independent Variables

Table 1 shows the descriptive statistics of the independent variables we included. These variables were as follows.

**Table 1:** The statistical parameters of the independent variables used in the analysis of the data from the 47 Japanese prefectures

Item	Daily internal medicine cost (US $, 1$=100yen)	Expansion rate (%)	Generic medicine replaced rate (%)	Proportion of elderly (%)
Data Number	47	47	47	47
Mean	2.61	63.1	24.1	12.9
Max	3.05	83.0	36.7	17.0
Min	2.23	35.2	18.9	8.7
S.D	0.17	10.6	2.71	2.14

Max = maximum, Min = minimum, S.D. = standard deviation

#### 2.2.1 The Expansion Rate (%)

This rate refers to the number of separation system upgrades among the administrative divisions in Japan; we obtained these data for the period of April 2011 to March 2012 by the JPS (2011).

#### 2.2.2 The Generic Medicine Replacement Ratio (%)

These data were taken from the JMHLW website—specifically, they were based on trends in the composition of medical expenses (for computation processing) in 2011 (Ministry of Health, Labour and Welfare, Insurance Bureau Security Research Division, 2011). From these data, we calculated the rate of generic medicines replaced by brand name medicines (hereafter, the generic medicine replacement ratio) for each prefecture. These data are exact because they are based on universal health care and medical insurance (which covers 99.9% of Japanese medical care) data.

#### 2.2.3 The Proportion of the Elderly (%)

The elderly were defined as people 75 years or older. We obtained data on the 75 years or older age group in the population (hereafter, the proportion of the elderly) for the fiscal year 2011 from the JSB website (Ministry of Internal Affairs and Communications Statistics Bureau, 2011). The proportion of the elderly for each prefecture is based on the city and district population data (as of October 1, 2011) of the national census conducted by the Ministry of Internal Affairs and Communications Statistics Bureau.

### 2.3 Data Analysis

A multiple regression analysis was conducted using the daily internal medicine cost per prescription (hereafter, daily internal medicine cost) as the dependent variable and the expansion rate, generic medicine replacement ratio, and proportion of the elderly as independent variables. All statistical analyses were performed by Excel Statistics 2010 (Society Information Service Co., Japan).

## 3. Results

The number of observations = the number of Japanese prefectures.

Table 2 shows the results of the multiple regression analysis. The results revealed a significant partial correlation for the expansion rate (X_1_) and the generic medicine replacement ratio (X_2_) for the items included in the analysis. A correlation analysis between daily medicine charge and the independent variables yielded the following regression functions:

**Table 2-1:** The result of multiple correlation analysis for daily internal medicine cost

Item	Daily internal medicines cost
Number of observations	47
Multiple correlation coefficient R	0.712
Coefficient of determination	0.507
*P-value*	>0.0001
*F-value*	14.72
*F (0.95)*	2.82

**Table 2-2:** The result of multiple correlation analysis for each indipendent variale

Item	Expansion rate (%)	Generic medicine replaced rate	Proportion of elderly (%)	Constant
Regression coefficient	-0.0070	-0.0290	0.0116	3.5990
Standard regression coefficient	-0.447	-0.474	0.150	-
Partial correlation coefficient	-0.535	-0.557	0.208	-
*P-value*	>0.001	>0.0001	*P*=0.17	-

Y_1_ = -0.0070X_1_^*^−0.0290X_2_^*^+0.0116X_3_+3.59, R = 0.712, P < 0.0001 (1)

where the variables are defined as follows:

Y_1_ = the daily internal medicine cost, X_1_ = the expansion rate, X_2_ = generic medicine replacement ratio, and X_3_ = the proportion of the elderly. The asterisk (*) indicates that the multiple regression analysis showed a significant partial correlation for these items. The results were rounded to the fourth decimal place.

## 4. Discussion

We found that daily internal medicine costs had a significant multiple correlations with two of the three factors hypothesized to influence medicine costs—expansion rate and generic medicine replacement ratio, which were examined for the first time in this study.

We observed a significant negative correlation for the relationship between expansion of the separation system and medicine costs. This finding indicates that the separation system in Japan might reduce daily medicine costs.

We observed a strong, positive multiple regression coefficient for daily internal medicine cost. Thus, we found that the separation system had a constant effect in reducing medicine costs; this is especially important, considering that internal medicine costs are 84% of the total outpatient medicine cost in Japan (Ministry of Health, Labour and Welfare, Insurance Bureau Security Research Division, 2011). Although this study did not determine why the medicine costs decreased with the increased expansion rate, there are some possible explanations we can discuss.

The separation of prescriptions and medicine sales enables doctors to refrain from prescribing specific medicines, as they are no longer personally interested in drug-related profit margins for their institutions or themselves. Moreover, many doctors believe that prescriptions will be reviewed by pharmacists, who are experts in dispensing medicines, unlike the doctors themselves. In addition, because doctors are not controlled by the stock available in their own institutions, they are free to prescribe any medication and can, therefore, choose less expensive medicines.

Very little data exist regarding the constant effect of the separation system on medicine cost reduction. However, we were able to identify a fixed effect here because the separation system is not mandatory in Japan; instead, it is the choice of the prescribing doctors in the medical institutions. Consequently, we were able to calculate the daily medicine costs data under various expansion rates and the effect of the separation system on medicine costs.

### 4.2 Relationship between Daily Internal Medicine Costs and Generic Medicine Replacement Ratio

We observed a statistically significant negative partial correlation between the generic medicine replacement ratio and daily internal medicine costs. Using generic medicines to reduce medicine costs is a well-known practice. The magnitude of the partial correlation coefficient for the generic medicine replacement ratio was similar to that of the expansion rate. Thus, with regard to reducing medicine costs in Japan, the separation system is as important as the use of generic medicines.

### 4.3 Relationship between Daily Internal Medicine Costs and Proportion of the Elderly

We observed a positive, non-significant partial correlation for the ratio of the elderly and internal medicine cost. Though this result seems consistent with the finding that medicine costs for the 75 years or older population accounts for 35% of national medicine costs, the proportion of the elderly did not have a strong effect on outpatient internal medicine costs. This is possibly because seriously ill patients are rapidly hospitalized in Japan; consequently, the medicine costs of outpatients would be lower than those of inpatients.

## 5. Conclusions

The results suggest that the separation system is as effective in reducing medicine costs as generic medicine usage. In light of the effects of the separation system on medical economic efficiency, the separation system should be expanded, especially in Asian countries in which the system is underdeveloped.
